# Cost of COVID-19 vaccine delivery in nine States in Nigeria via the U.S. Government Initiative for Global Vaccine Access

**DOI:** 10.1186/s12913-024-11645-1

**Published:** 2024-10-14

**Authors:** Dave Haeyun Noh, Roopa Darwar, Belinda V. Uba, Shiva Gab-deedam, Stella Yani, Akolade Jimoh, Ndadilnasiya Waziri, Joshua David, Babatunde Amoo, Sunday Atobatele, Janada Dimas, Rhoda Fadahunsi, Sidney Sampson, Edwin Simple, Gideon Ugbenyo, Margeret Wisdom, Adeyelu Asekun, Sarah W. Pallas, Hadley Ikwe

**Affiliations:** 1 Global Immunization Division, Global Health Center, Centers for Disease Control and Prevention, Atlanta, GA USA; 2 African Field Epidemiology Network (AFENET) Nigeria, Abuja, Nigeria; 3 Sydani Group Nigeria, Abuja, Nigeria; 4 Sydani Institute for Research and Innovation (SIRI), Abuja, Nigeria; 5 Global Immunization Division, Global Health Center, Centers for Disease Control and Prevention, Abuja, Nigeria

**Keywords:** Nigeria, COVID-19, Vaccine, Costs

## Abstract

**Background:**

In 2022, the U.S. Centers for Disease Control and Prevention collaborated with implementing partners, African Field Epidemiology Network and Sydani Group, to support COVID-19 vaccination efforts in Nigeria. To characterize the costs of COVID-19 vaccination, this study evaluated financial costs per dose for activities implemented to support the intensification campaign for COVID-19 vaccination.

**Methods:**

This retrospective evaluation collected secondary data from existing expenditure and programmatic records on resource utilization to roll out COVID-19 vaccination during 2022. The study included incremental financial costs of the activities implemented to support an intensification campaign for COVID-19 vaccination across nine states and six administrative levels in Nigeria from the perspective of the external donor (U.S. Government). Costs for vaccines and injection supplies, transport of vaccines, and any economic costs, including government in-kind contributions, were not included. All costs were converted from Nigerian Naira to 2022 U.S. Dollars (US$).

**Results:**

The estimated financial delivery cost of the COVID-19 vaccination intensification campaign was US$0.84 per dose (total expenditure of US$6.29 million to administer 7,461,971 doses). Most of the financial resources were used for fieldwork activities (86%), followed by monitoring and supervision activities (8%), coordination activities (5%), and training-related activities (1%). Labor (58%) and travel (37%) were the resource inputs that accounted for the majority of the cost, while shares of other resource inputs were marginal (1% for each). Most labor costs (79%) were spent on payments for mass vaccination campaign teams, including pay-for-performance incentives. By administrative level, the largest share of costs (46%) was for pay-for-performance incentives at the community, health facility, or campus levels combined, followed by local government area level (24%), community level only (15%), state level (9%), national level (3%), campus level only (1%), and health facility level only (< 1%).

**Conclusions:**

Findings from the evaluation can help to inform resources needed for vaccination activities to respond to future outbreaks and pandemics in resource-limited settings, particularly to reach new target populations not regularly included in routine childhood immunization delivery.

## Background

To mitigate the proliferation of severe acute respiratory syndrome coronavirus 2 (SARS-CoV-2), many countries announced intensive efforts to vaccinate their populations against the disease in 2021 and 2022, during the height of the coronavirus disease 2019 (COVID-19) pandemic [1]. However, low- and middle-income countries (LMICs) confronted challenges in procuring and delivering effective COVID-19 vaccines to all eligible populations due to concerns regarding vaccine accessibility and affordability [2].

The United States (U.S.) Government committed to global efforts to vaccinate 70% of the population in every country against SARS-CoV-2 in 2022 by helping countries receive, distribute, and administer doses of COVID-19 vaccines [3]. Led by the U.S. Agency for International Development in close partnership with the U.S. Centers for Disease Control and Prevention (CDC) and other U.S. Government agencies, the U.S. Government Initiative for Global Vaccine Access (Global VAX) aimed to expand assistance and improve international coordination to identify and overcome vaccine access barriers, with an emphasis on scaling up vaccination support in the African Region [3, 4].

In Nigeria, the U.S. Mission Nigeria and CDC utilized their existing networks of partners in-country to support intensified vaccination efforts following the provision of Global VAX resources in 2022 [5]. CDC collaborated with two implementing partners in Nigeria, namely the African Field Epidemiology Network (AFENET) and Sydani Group (Sydani), to support vaccination efforts in nine states. The U.S. Government, through Global VAX, invested US$ 6.29 million to support vaccination activities in 2022, such as coordination meetings, trainings, mass vaccination campaigns at various locations, outreach vaccination for disadvantaged groups (e.g., at HIV clinics, correctional facilities, internally displaced person camps, and nomadic settlements), and monitoring and supervision. Among the innovative features of this vaccination initiative was the use of performance-based financial incentives for local health care workers to deliver vaccination, i.e., pay-for-performance (P4P), in which health care providers were remunerated based on the number of individuals vaccinated per day.

The P4P approach has been widely used in health programs to attempt to increase the quality of care provided by health care providers since the late 1990s, but its impacts on a COVID-19 vaccination program have not been evaluated [6–12]. By aligning the interests of health care providers with those of patients and society, P4P approaches generally aim to ensure that health care workers provide optimal levels of effort by reducing the “know-do” gap [13]. Rewarding providers for achieving prespecified performance targets may also be an efficient approach if it can save monitoring costs [14].

Currently, case studies from Botswana, Côte d’Ivoire, Democratic Republic of the Congo, Kenya, Mozambique, and Uganda provide empirical evidence on COVID-19 vaccine delivery costs in the African Region, albeit with limited information on the cost of varied delivery strategies [15–21]. Other analyses in this topical space in low-resource settings include modeled estimates, empirical evidence, case studies in countries outside the African Region (i.e., Philippines and Vietnam; Bangladesh report forthcoming), and last mile delivery costs [21–23]. For instance, the costs of COVID-19 vaccine delivery in Mozambique were found to differ by phase of vaccine delivery, with higher costs per dose during initial phases with vaccine supply constraints and small target populations compared to lower costs during later phases with larger target populations due to efficiencies in reaching economies of scale [20]. The financial and economic costs of COVID-19 vaccine delivery in Côte d’Ivoire were estimated to be between those for the two phases in Mozambique [19, 20].

However, these empirical studies did not include intensified outreach for disadvantaged populations or P4P incentives. In addition, no evaluation has been conducted on COVID-19 vaccine delivery costs in Nigeria, the most populous country in the African Region. To better characterize the costs of COVID-19 vaccine delivery, this evaluation examined financial costs per dose for activities implemented to support the COVID-19 vaccination campaigns in nine CDC-supported states in Nigeria. This examination facilitated understanding of the composition of costs for intensive vaccination campaigns with large dose delivery volumes reaching novel, adult target populations not reached by routine childhood immunization delivery services. By identifying key delivery activities and the associated costs, findings from this study are expected to inform future investments needed to support mass vaccination activities in resource-limited settings, including rollout of new vaccines in response to future epidemics or pandemics.

## Methods

**Fig. 1:**
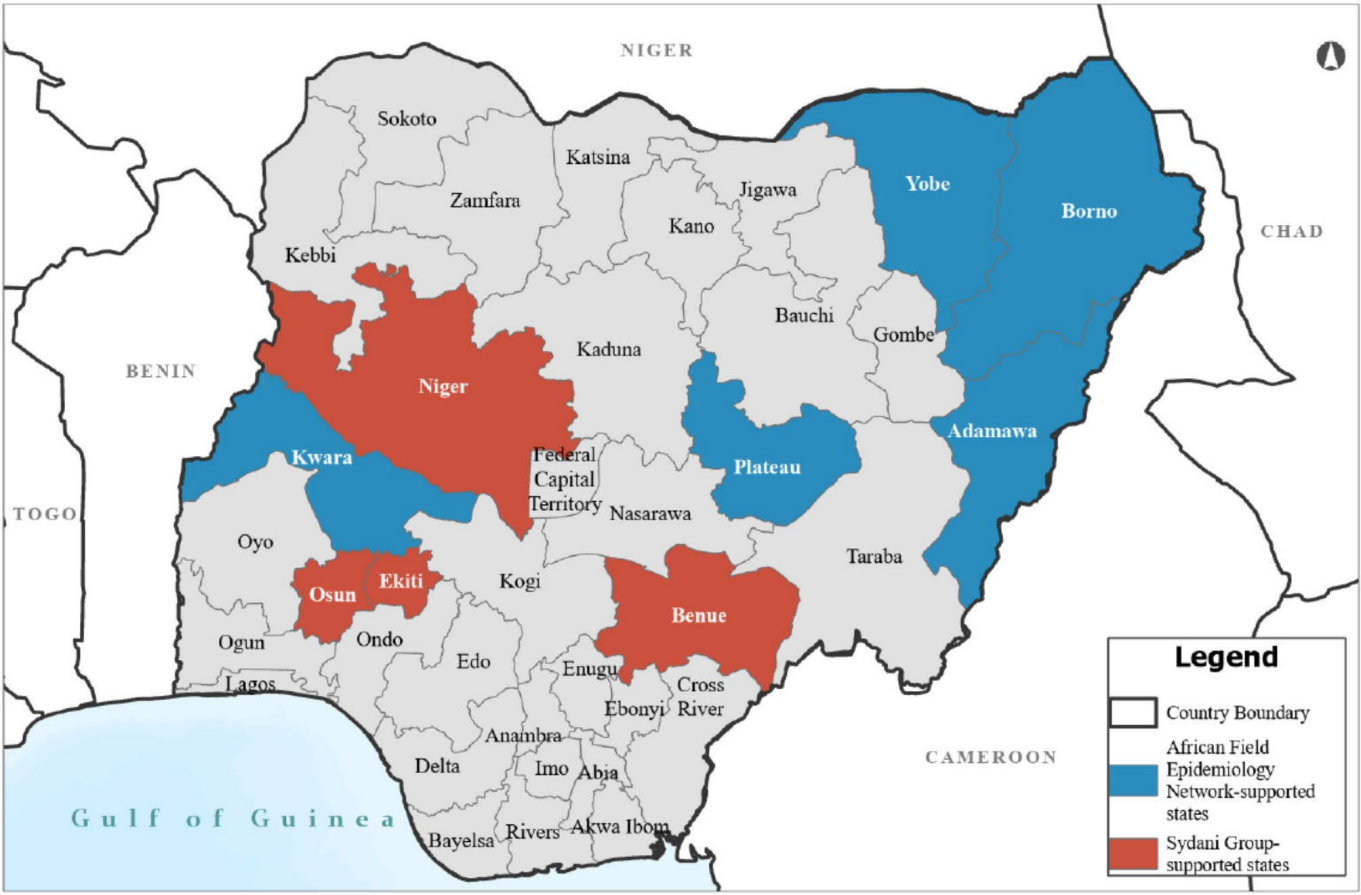
Nine States Supported by AFENET and Sydani Group. Data source: Environmental Systems Research Institute (Esri). Map developed by Geospatial Research, Analysis, and Services Program (GRASP); Agency for Toxic Substances and Disease Registry; U.S. Centers for Disease Control and Prevention

This retrospective evaluation used an ingredients-based approach to estimate the incremental financial costs (i.e., monetary outlays) of the COVID-19 vaccine delivery activities in Nigeria over the analytic horizon of 1 January 2022 through 31 December 2022, from the perspective of the external donor (U.S. Government). The evaluation includes nine CDC-supported states, for which “supported” indicates that the implementing partner received funding awards from CDC for COVID-19 vaccination delivery activities in the selected states (Table 1). The nine states, with a total of 23,237,360 individuals eligible for COVID-19 vaccines, were purposively selected by AFENET and Sydani for COVID-19 vaccine delivery out of the 36 states and the Federal Capital Territory of Nigeria (Fig. 1). Delivery of COVID-19 vaccines in five states (Adamawa, Borno, Kwara, Plateau, and Yobe) was supported by AFENET, while that for four other states (Benue, Ekiti, Niger, Osun) was supported by Sydani.

**Table 1:** Implementing partner timeframe of support by State and target population number

**Implementing partner**	**State**	**Support start date**	**Support end date**	**Target population**
AFENET	Adamawa	January 2022	December 2022	2,515,768
AFENET	Kwara	January 2022	November 2022	1,912,003
AFENET	Borno	July 2022	December 2022	3,356,269
AFENET	Plateau	July 2022	December 2022	2,596,406
AFENET	Yobe	July 2022	December 2022	1,848,791
Sydani	Benue	May 2022	September 2022	3,241,297
Sydani	Osun	May 2022	September 2022	2,568,226
Sydani	Ekiti	June 2022	September 2022	1,948,227
Sydani	Niger	June 2022	September 2022	3,250,373

Incremental financial costs associated with the vaccination activities were evaluated to examine the COVID-19 vaccination delivery cost. Secondary data were collected from the two implementing partners from existing expenditure and programmatic records on resource utilization to roll out COVID-19 vaccines in the supported states using a standardized Excel template, capturing all financial costs of these partners to implement the intervention (i.e., a census of financial costs; no sampling was performed). Data on existing in-kind resources contributed by government or other partners were not available to estimate economic costs due to the retrospective nature of this ex-post evaluation. The financial data of implementing partners were collected using the Excel tool to capture unit prices and quantities of resource inputs used for COVID-19 vaccine delivery activities. All cost items were mapped against the three dimensions for this evaluation: program activity, resource input, and administrative level. All costs were converted from Nigerian Naira to 2022 U.S. Dollars (US$) using the Central Bank of Nigeria average monthly exchange rates throughout 2022 [24].

This evaluation categorized COVID-19 vaccine delivery activities as coordination, fieldwork, monitoring, or training. Coordination activities consisted of administrative, financial, and operational activities, as well as those for project oversight, facilitation of stakeholder engagements, and deployment planning. Fieldwork activities included social mobilization efforts, transport of vaccine to vaccination delivery sites, and vaccination services during university or college “campus storms” and “market storms” (i.e., where multiple vaccination teams “stormed” a campus or marketplace to provide easily accessible vaccination throughout the campus/market during a time-limited period), in addition to outreach vaccination sessions at HIV clinics, correctional facilities, internally displaced person camps, and nomadic settlements. Monitoring activities consisted of all supportive supervision activities for mass vaccination teams and weekly performance review meetings. Training included both in-person and virtual training activities organized for health care workers prior to mass vaccination campaigns.

Costs were concurrently mapped by resource input. These resources included labor, equipment, supplies, travel, transport, rent, utilities, contracts, other. Labor costs included those for management support team members, mass vaccination team community mobilizers, national consultants, and other additional labor contracted for specific terms of reference by implementing partners for COVID-19 vaccination scale-up activities. Focal points from each implementing partner interviewed all staff on the mass vaccination campaign initiative to collect self-reported shares of staff time on the initiative. Mass vaccination campaign team members were remunerated based on the number of individuals vaccinated per day under the P4P payment scheme. Equipment included items with a useful life of more than one year or with multi-use potential, such as computers, phones, printers, and furniture, purchased by implementing partners for COVID-19 vaccination scale-up activities. Supplies included materials, consumables, and supplies with a useful life of less than one year procured by implementing partners for COVID-19 vaccination scale-up activities (e.g., printing of forms, booklets, explanatory materials, notebooks, pens, training materials). Travel costs included those for the travel or lodging of personnel, such as for per diems, allowances, vehicle purchase, vehicle rental, fuel, driver hire, and public transit fare. Transport costs included those for the travel of items or goods, such as for vehicle purchase, vehicle rental, fuel, driver hire, and public transit fare. Both travel costs and transport costs include similar types of resource inputs, but are differentiated by what was transported (people for travel costs, goods for transport costs). Rent costs included those for renting office space and renting venues for COVID-19 vaccination scale-up activities (e.g., training, meetings, other events). Costs of internet service provision, mobile phone service, electricity, and any other utilities used for COVID-19 vaccination scale-up activities were classified under utilities. Contracts included costs for contracted services, such as information technology, security, cleaning, or other professional services. Other costs encompassed costs for any items and activities not otherwise categorized.

Costs were also mapped by administrative level, including national, state, local government area (LGA), community, health facility, and campus. Costs could not be mapped to the fourth administrative level in Nigeria, the ward level between LGA and community levels, as data were not available at that level. Activities across multiple states were categorized as national level activities if the cost components for the multi-state activities could not be disaggregated by state by the implementing partners.

This analysis excluded costs that were not paid by the implementing partners, such as the costs of vaccines, injection supplies, and vaccine transportation from central medical stores to health facilities. Economic costs, including government in-kind contributions were excluded from the analysis. Costs of patient time to receive vaccination, CDC in-kind staff time, and conducting this cost analysis were also excluded.

To estimate the per-dose cost, records on the number of doses delivered daily by each vaccination team from each supported state were collected for the analysis. Existing dose data was recorded in a tabular format in Microsoft Excel after de-identifying mass vaccination campaign team information. The per-dose cost was estimated by dividing the aggregated incremental costs by the total number of doses administered in the CDC-supported states.

## Results

The estimated financial cost of COVID-19 vaccine delivery in the nine CDC-supported states in Nigeria was US$0.84 per dose in 2022. Total expenditures of US$6.29 million were incurred by implementing partners to support vaccination activities to administer 7,461,971 doses.

**Fig. 2:**
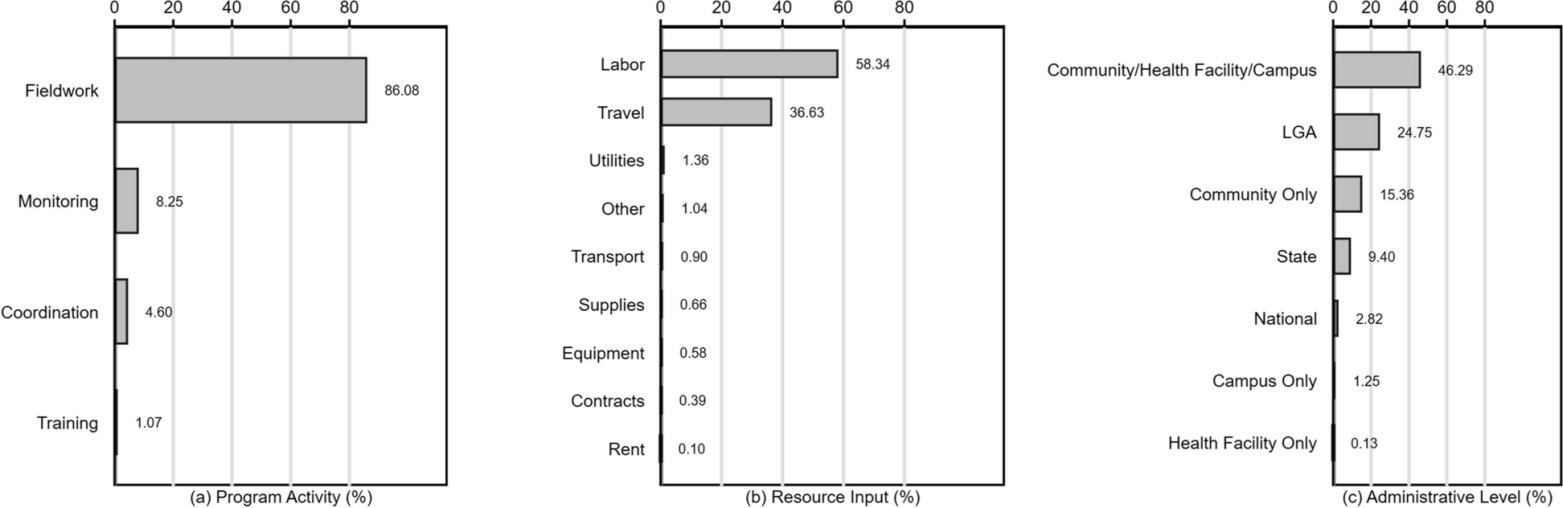
Financial cost distribution by dimension

Panel (a) of Fig. 2 exhibits that fieldwork claimed the largest share of financial costs among all program activities. Approximately 86.08% of the total costs (US$0.73 out of US$0.84 per dose) was incurred to support fieldwork, including vaccination campaigns and outreach vaccination sessions at various locations, across the nine states. Compared to the fieldwork costs, relatively small fractions of costs were spent on monitoring (8.25% or US$0.07 per dose), coordination (4.60% or US$0.04 per dose), and training (1.07% or US$0.01 per dose).

Panel (b) shows cost distribution across nine resource inputs. Of those, labor costs accounted for the largest share of financial costs (58.34% or US$0.49 per dose). Labor costs included implementing partner staff salaries and payments for health care workers, including P4P incentives. Health care workers received additional payments depending on the number of COVID-19 vaccine doses their respective teams administered per day; of the US$0.49 labor costs per dose, 79.34% (US$0.39) were paid to health care workers. The second largest resource input, at 36.63% of the financial costs (US$0.31 per dose), were travel-related costs for various vaccination activities. Labor and travel costs accounted for approximately 94.97% of the financial costs, while shares of other resource inputs were marginal (1.36% for utilities, 1.04% for other, 0.90% for transport, 0.66% for supplies, 0.58% for equipment, 0.39% for contracted services, and 0.10% for rent).

Panel (c) of Fig. 2 presents the financial cost distribution by administrative level. The largest share (46.29%) of the financial costs was allocated to payments for health care workers involved in mass vaccination campaigns and outreach vaccination sessions at community, health facility, and campus levels combined; however, the specific distribution of these costs across each of these levels could not be estimated due to data limitations. The next largest share of financial costs (24.75%) was incurred at LGA level (US$0.21 per dose), followed by community-only (15.36% or US$0.13 per dose), state (9.40% or US$0.08 per dose), national (2.82% or US$0.02 per dose), and campus-only levels (1.25% or US$0.01 per dose). Financial costs at the health facility level (not including P4P incentives) were less than 1% (less than US$ 0.01 per dose).

Table 2 illustrates per-dose financial cost by program activity at each administrative level. Coordination-related costs were reported primarily at the state (US$0.02 per dose or 47.44% of the coordination costs) and at the national levels (US$0.01 per dose or 35.08% of the coordination costs). While the amounts were not significant, most of the training costs were incurred at the LGA level (US$0.01 per dose or 67.48% of the training costs). Fieldwork costs were reported across all levels. Of the US$0.73 fieldwork activity costs per dose, more than half of the expenses were incurred for health care worker payment for mass vaccination campaigns and outreach vaccination sessions at communities, health facilities, and campuses (US$0.39 per dose or 53.78% of the fieldwork costs), followed by the LGA level (US$0.17 per dose or 22.82% of the fieldwork costs) and the community level (US$0.13 per dose or 17.85% of the fieldwork costs). Finally, monitoring costs were reported at the national (US$0.01 per dose or 10.31% of the monitoring costs), state (US$0.03 per dose or 46.09% of the monitoring costs), and LGA levels (US$0.03 per dose or 43.59% of the monitoring costs) only.

**Table 2:** Financial cost per dose – program activity by administrative level

**Unit: 2022 US$ (%)**		**Administrative level**							
		**National**	**State**	**LGA**	**Community only**	**Health facility only**	**Campus only**	**Community/health facility/campus**	**Total**
**Program Activity**	**Coordination**	$0.01 (1.62)	$0.02 (2.18)	$0.01 (0.79)	-	-	< $0.01 (0.01)	-	$0.04 (4.60)
	**Training**	< $0.01 (0.09)	< $0.01 (0.26)	$0.01 (0.72)	-	-	-	-	$0.01 (1.07)
	**Fieldwork**	< $0.01 (0.27)	$0.03 (3.15)	$0.17 (19.64)	$0.13 (15.36)	< $0.01 (0.13)	$0.01 (1.24)	$0.39 (46.29)	$0.73 (86.08)
	**Monitoring**	$0.01 (0.85)	$0.03 (3.80)	$0.03 (3.60)	-	-	-	-	$0.07 (8.25)
	**Total**	$0.02 (2.82)	$0.08 (9.40)	$0.21 (24.75)	$0.13 (15.36)	< $0.01 (0.13)	$0.01 (1.25)	$0.39 (46.29)	$0.84 (100.00)

Table 3 shows per-dose financial cost by program activity and resource input. More than half of coordination costs were used for labor (US$0.03 per dose or 67.51% of the coordination cost), while travel accounted for 9.95% of the coordination costs (< US$0.01 per dose). Most of the training costs were used for travel (63.32% or US$0.01 per dose), while supplies, rent, utilities, and contracts each accounted for less than 10% of the training costs. Fieldwork costs were primarily for labor (US$0.43 per dose or 59.46% of the fieldwork costs) and travel (US$0.27 per dose or 36.81% of the fieldwork costs). Similarly, these two resource inputs contributed the most to the monitoring costs (49.11% and 46.14% of the monitoring cost, respectively).

**Table 3:** Financial cost per dose – program activity by resource input

**Unit: 2022 US$ (%)**		**Resource input**									
		**Labor**	**Equipment**	**Supplies**	**Travel**	**Transport**	**Rent**	**Utilities**	**Contracts**	**Other**	**Total**
**Program Activity**	**Coordination**	$0.03 (3.11)	-	-	< $0.01 (0.46)	-	-	$0.00 (0.00)	-	$0.01 (1.04)	$0.04 (4.60)
	**Training**	-	-	< $0.01 (0.06)	$0.01 (0.68)	-	< $0.01 (0.10)	< $0.01 (0.15)	< $0.01 (0.08)	-	$0.01 (1.07)
	**Fieldwork**	$0.43 (51.18)	< $0.01 (0.58)	$0.01 (0.60)	$0.27 (31.69)	$0.01 (0.90)	-	$0.01 (1.13)	-	-	$0.73 (86.08)
	**Monitoring**	$0.03 (4.05)	-	-	$0.03 (3.81)	-	-	< $0.01 (0.08)	< $0.01 (0.31)	-	$0.07 (8.25)
	**Total**	$0.49 (58.34)	< $0.01 (0.58)	$0.01 (0.66)	$0.31 (36.63)	$0.01 (0.90)	< $0.01 (0.10)	$0.01 (1.36)	< $0.01 (0.39)	$0.01 (1.04)	$0.84 (100.00)

Finally, Table 4 presents the financial cost per dose by resource input at each administrative level. Most of the labor costs, amounting to US$0.39 per dose or 79.34% of total labor costs, were incurred at the community/health facility/campus levels combined for vaccination team P4P compensation, while about 8% of the labor costs were allocated at both the state and community-only levels. Equipment costs were reported at the community (< US$0.01 per dose) and campus (< US$0.01 per dose) levels for community engagement, sensitization, and mobilization activities. Supply costs were reported for the LGA (< US$0.01 per dose), health facility (< US$0.01 per dose), and campus (< US$0.01 per dose) levels only. Travel costs were reported across all administrative levels except for the health facility level. The largest portion of the travel costs was spent by implementing partners to support LGA-level activities (US$0.19 per dose or 62.24% of the travel costs), with 24.84% of the travel costs (US$0.08 per dose) were devoted to community-level activities. Transport costs of goods were marginal and reported at the national and community levels only.

**Table 4:** Financial cost per dose – resource input by administrative level

**Unit: 2022 US$ (%)**		**Administrative level**							
		**National**	**State**	**LGA**	**Community only**	**Health facility only**	**Campus only**	**Community/health facility/campus**	**Total**
**Resource Input**	**Labor**	$0.01 (0.95)	$0.04 (4.63)	$0.01 (1.58)	$0.04 (4.89)	-	-	$0.39 (46.29)	$0.49 (58.34)
	**Equipment**	-	-	-	< $0.01 (0.50)	-	< $0.01 (0.08)	-	< $0.01 (0.58)
	**Supplies**	-	-	< $0.01 (0.19)	-	< $0.01 (0.13)	< $0.01 (0.34)	-	$0.01 (0.66)
	**Travel**	< $0.01 (0.45)	$0.03 (3.46)	$0.19 (22.80)	$0.08 (9.10)	-	$0.01 (0.82)	-	$0.31 (36.63)
	**Transport**	< $0.01 (0.03)	-	-	$0.01 (0.87)	-	-	-	$0.01 (0.90)
	**Rent**	< $0.01 (0.09)	-	< $0.01 (0.02)	-	-	-	-	< $0.01 (0.10)
	**Utilities**	< $0.01 (0.28)	$0.01 (1.00)	< $0.01 (0.09)	-	-	-	-	$0.01 (1.36)
	**Contracts**	-	< $0.01 (0.31)	< $0.01 (0.08)	-	-	-	-	< $0.01 (0.39)
	**Other**	$0.01 (1.04)	-	-	-	-	-	-	$0.01 (1.04)
	**Total**	$0.02 (2.82)	$0.08 (9.40)	$0.21 (24.75)	$0.13 (15.36)	< $0.01 (0.13)	$0.01 (1.25)	$0.39 (46.29)	$0.84 (100.00)

## Discussion

This evaluation estimated a financial cost of US$ 0.84 to deliver one dose of COVID-19 vaccine in these nine states in Nigeria using mass campaign and outreach vaccination strategies and provision of monetary incentives to health care workers.

The per-dose financial delivery cost in Nigeria is lower than that for the small target populations estimate from the Mozambique study (US$ 0.96), but higher than that for that the larger target population estimate from the Mozambique study (US$ 0.43) [20]. Also, the per-dose financial delivery cost in Nigeria is higher than that in Côte d’Ivoire (US$ 0.16) [19]. However, the results from the studies should be compared with a caveat because the scope of the included activities varies by study. One of the primary differences from this evaluation in Nigeria as compared to other studies concerns the inclusion of costs of intensified outreach efforts for disadvantaged populations (e.g., those in HIV clinics, correctional facilities, internally displaced person camps, and nomadic settlements), which were excluded from the other existing empirical studies. Although data on dose delivery by site were not available, intensified outreach and vaccination sessions at smaller scale sites for disadvantaged populations may increase the costs due to distribution of fixed costs of delivery over a smaller target population, as well as potential logistical challenges requiring additional operational costs for more complex coordination, monitoring, and supervision.

Secondly, our findings showed that a significant amount of funds were invested in payments made to health care workers (US$0.39 out of US$ 0.84 per dose), including P4P incentives. In this study, it was not possible to evaluate the effects of P4P on vaccination coverage due to the absence of a prospective study design to isolate the effects of the financial incentives under pandemic response conditions. Previous studies provide mixed empirical evidence regarding the potential for P4P approaches to improve immunization coverage for childhood vaccines [25–28]. The direction of the effect of P4P on the number of doses administered is theoretically ambiguous. If the financial reward incentivizes increases in the marginal benefits of the extra efforts while its marginal costs remain the same for health care workers, the quality of services (i.e., the number of doses administered per day) is expected to be improved in line with the existing studies for other health programs. Conversely, P4P may have negative effects on vaccination coverage if the financial incentives crowd out the intrinsic motivations of health care workers [29]. Further research to examine the effects of P4P on the coverage of COVID-19 vaccines is needed to understand the potential value of this approach during a potential outbreak or pandemic in the future.

This campaign with a new vaccine targeting a novel population during an emergency response to a pandemic required costs similar to those for other preventive immunization campaigns targeting children in Nigeria. For instance, a measles campaign in Anambra state in Nigeria resulted in an average operational cost per dose (excluding vaccine costs) of 2022 US$1.10 [30, 31]. Both integrated yellow fever and meningitis A and standalone yellow fever campaigns in various states in Nigeria during 2019–2020 required approximately 2022 US$0.41 per dose and 2022 US$0.34–$0.40, respectively, in financial costs (excluding vaccine costs) [31, 32].

While the COVID-19 mass vaccination campaign in nine states in Nigeria was not exceedingly more costly than other, more common immunization campaigns in-country, an immunization campaign targeting an adult population in an emergency response is unlikely to be required frequently. Accordingly, the model of this initiative and its associated incremental financial costs is not assumed or proposed to be a sustainable option for vaccinating individuals with antigens in Nigeria’s National Immunization Program on a routine basis. The costs of this donor-funded intervention conducted through non-governmental organization implementing partners may differ in both costs and effects from similar interventions implemented through government and funded with domestic government resources alone. The cost-effectiveness of different vaccination modalities may vary by antigen and across target populations and subnational settings in Nigeria.

Our results should be interpreted in light of several limitations. Although this evaluation has accounted for all financial costs associated with implementing the activities, it is important to acknowledge that the absence of other data on the value of in-kind resources contributed by government and other partners, which limits our ability to completely understand the resources required for effective vaccination delivery as part of pandemic responses. For example, the labor time costs of government health care workers who served as vaccinators are not included in our estimates because economic costs of government staff time are beyond the scope of this evaluation. However, any per diems or allowances paid to government health care workers by the implementing partners for participation in the vaccination activities are included in our financial cost estimates from the implementing partner perspective. Second, we could not isolate the impact of the intervention package from other confounding factors, such as time trend effects, due to the absence of the comparison group. While 7,461,971 doses were administered by the vaccination campaigns organized by the CDC-supported implementing partners, we could not attribute the total number of doses to the activities of implementing partners since certain individuals who received vaccinations might have sought and received vaccinations even in the absence of the intervention of the implementing partners. Third, there is a possibility of recall bias in the allocation of implementing partner personnel time across activities; however, the direction of any such bias across program activities is unknown and this would not affect the total incremental financial cost. Finally, the findings from this evaluation have limitations in the generalizability to other settings in that the nine states were purposively sampled.

## Conclusions

This evaluation estimated the incremental financial delivery cost per dose of COVID-19 vaccine in 2022 via various intensification and mass campaign activities in nine states in Nigeria supported by CDC. These detailed costs of COVID-19 vaccination activities provide empirical evidence on the costs of these strategies as implemented in the midst of the COVID-19 pandemic when alternative evaluation designs (e.g., a randomized controlled trail) were not feasible. The estimates from this evaluation are expected to provide data to inform vaccination strategy options for global and national immunization program managers for future outbreak and pandemic responses when new target populations may need to be rapidly reached with new vaccines or other countermeasures, and to facilitate policy decisions in resource-limited settings.

## Data Availability

Data will not be publicly available as cost data includes confidential procurement information for implementing partners.

## Abbreviations

AFENET: African Field Epidemiology Network
CDC: (U.S.) Centers for Disease Control and Prevention
COVID-19: Coronavirus disease 2019
Global VAX: Initiative for Global Vaccine Access
LGA: Local government area
LMICs: Low- and middle-income countries
P4P: Pay-for-performance
SARS-CoV-2: Severe acute respiratory syndrome coronavirus 2
Sydani: Sydani Group
U.S.: United States
